# Himawari-8 Satellite Based Dynamic Monitoring of Grassland Fire in China-Mongolia Border Regions

**DOI:** 10.3390/s18010276

**Published:** 2018-01-18

**Authors:** Li Na, Jiquan Zhang, Yulong Bao, Yongbin Bao, Risu Na, Siqin Tong, Alu Si

**Affiliations:** 1 School of Environment, Northeast Normal University, Changchun 130024, China; lin152@nenu.edu.cn (L.N.); baoyb924@nenu.edu.cn (Y.B.); tongsq223@nenu.edu.cn (S.T.); alusi007@126.com (A.S.); 2 Key Laboratory for Vegetation Ecology, Ministry of Education, Changchun 130024, China; 3 Collage of Geography, Inner Mongolia Normal University, Hohhot 010022, China; baoyulong@imnu.edu.cn; 4 School of Geographical Sciences, Northeast Normal University, Changchun 130024, China; nars582@nenu.edu.cn

**Keywords:** Threshold Algorithm, visual interpretation, Himawari-8 satellite, grassland fire, China-Mongolia border regions

## Abstract

In this study, we used bands 7, 4, and 3 of the Advance Himawari Imager (AHI) data, combined with a Threshold Algorithm and a visual interpretation method to monitor the entire process of grassland fires that occurred on the China-Mongolia border regions, between 05:40 (UTC) on April 19th to 13:50 (UTC) on April 21st 2016. The results of the AHI data monitoring are evaluated by the fire point product data, the wind field data, and the environmental information data of the area in which the fire took place. The monitoring result shows that, the grassland fire burned for two days and eight hours with a total burned area of about 2708.29 km^2^. It mainly spread from the northwest to the southeast, with a maximum burning speed of 20.9 m/s, a minimum speed of 2.52 m/s, and an average speed of about 12.07 m/s. Thus, using AHI data can not only quickly and accurately track the dynamic development of a grassland fire, but also estimate the spread speed and direction. The evaluation of fire monitoring results reveals that AHI data with high precision and timeliness can be highly consistent with the actual situation.

## 1. Introduction

Grassland fire prevention, detection, and monitoring are key economic and public safety concerns in many parts of the world [1]. Grassland fires occur frequently in China. The Inner Mongolia Autonomous Region is located between China and Mongolia, at the junction of the two countries. The border is 3193 km long, accounting for 68% of the total length of the border line between China and Mongolia [2,3]. Mongolia is sparsely populated, has weak firefighting capabilities, as well as less precipitation in spring and autumn, dry weather, and a large number of gale-force wind days. As a result, grassland fires occurring in the China-Mongolia border regions are often spread into Inner Mongolia, China by the wind. Statistics show that in 1994–1997, the cross-border fires in Mongolia caused more than 20 grassland fires in China. In addition, there were six fire threats to border regions of China. In particular, the fires outside the country’s borders have been more frequent and larger since 2000, and the threat to China has gradually increased. Among them, cross-border fires occurring in 2000, 2003, 2005, 2009, 2011, 2012 and 2014 have had a serious impact [4]. Fire risk and potential danger have generally been associated with stand fuel characteristics, topographical features, land use, weather, climate, and social factors [5], hence the occurrence has a certain uncertainty and randomness. The rapid spread of grassland fires makes alleviation and rescue difficult, and it can seriously endanger grassland resources [6]. Therefore, the rapid and dynamic monitoring of grassland fires is indispensable in the work of firefighting, and also plays an important role in early warning and assessment.

Satellite-based remote sensing with a fast imaging cycle, high spatiotemporal resolution and wide coverage provides a new technology for fire monitoring. It can continuously monitor the dynamic development process of grassland fires, and provide a more accurate fire location and burned area. In the early 1960s, aeronautical infrared detection was used to monitor large-scale fire information. However, the international monitoring of fire by remote sensing began in the late 1970s and early 1980s [7]. From Planck’s blackbody radiation law, it is known that the maximum radiation wavelength is within the mid-infrared spectral range. Hence, we can inference the fire points can be detected by the much higher detector signals caused by the fire points in the mid-infrared channel compared to these of non-fire points [8,9]. Therefore, in the algorithms for identification of the burning fire point, the Threshold Model was developed-based on the Geostationary Operational Environmental Satellite (GOES) and National Oceanographic and Atmospheric Administration (NOAA) satellite’s Advanced Very High Resolution Radiometer (AVHRR) sensor image data [10,11], and the threshold value is mainly given to the bright temperature value of the channels 3 and 4 of the AVHRR data. Considering the fact that in different regions and at different times, there are different climate and vegetation factors, the Contextual Threshold method was further developed in 1990 based on the Threshold Model. In 2000, the threshold of combustibles (the amount of combustibles represented by NDVI_MVC_) was added to the identification of the fire point of the Contextual thresholds to further establish the Combustible Coverage model of fire point identification [12,13,14]. However, the Moderate-Resolution Imaging Spectroradiometer (MODIS), a medium-resolution imaging spectrometer carried by the Earth Observation System (EOS) satellite launched by the National Aeronautics and Space Administration (NASA) in 1999, has improved resolution and accuracy compared with the AVHRR. Good spatiotemporal and spectral resolution provides a higher application prospect in the field of forest and grassland fire monitoring [15]. The current MODIS active fire algorithm “version 4” [16] is the latest version of the initial algorithm with improvements made to detect smaller fires and reduce false detections [17,18]. With the popularization of remote sensing images, remote sensing data such as NPP/VIIRS, FY-3/VIRR and HJ-1A/B have been applied to the extraction of high temperature fire points [19,20,21]. In recent times, the emergence of a series of global fire point product data such as MOD14 and MYD14, FY-3/VIRR-GFR and NPP/VIIRS-Active have pushed the study of forest and grassland fires to a high point. Due to the characteristics of large areas and rapid development of grassland fires, the lower temporal resolution of the above data cannot meet the dynamic monitoring of its development process. The Advanced Himawari Imager (AHI) sensor data of the Himawari-8 Satellite that was officially launched by the Japan Meteorological Agency in October 2014 provides a temporal resolution that represents a new breakthrough in the field of remote sensing, covering an entire hemisphere every 10 min with a larger channel wavelength range. This is an important data source for monitoring the abrupt and a fast development of grassland fires. Hence, AHI data allows to monitor high dynamic fire events stretched out over a large area (like the grassland fires). However, because of the development of satellite data is new, it has not been popularized in the various fields of research. In the research of grassland fires, in 2015, bands 3, 4, and 6 of the Himawari-8 satellite data were used to monitor grassland fires by using an RGB color synthesis method [22], and the fire point was identified by assigning a threshold to the bright temperature value of band 7 in 2016 [23,24,25]. However, according to the characteristics of grassland fire, different regions and different time periods both affect the threshold of the fire point temperature. Through this research, provide the methods of monitoring fire point suitable for the regional characteristics for the grassland fire management department of the China and Mongolia border regions.

Hence, based on the result of the above research and the algorithm suitable for this study area was obtained by improving the Threshold Algorithm of predecessors. Then visual interpretation was used to acquire the burning fire points based on the color synthesis of the AHI sensor bands 7, 4, and 3. According to band 4, we can observe the changes of the burned and unburned area, so as to extract the burned area. Therefore, in this study, the improved Threshold Algorithm and visual interpretation are used to monitor the grassland fires occurring in the China-Mongolia border regions from April 19th to the 21st, 2016. The real-time wind field data, environmental information data, fire point product data and Landsat8/OLI higher resolution image data were used to evaluate the effect of the monitoring results. This work will provide the fastest and up-to-the-minute fire information extraction method for the early warning of cross-border fires and propose an effective scientific basis for firefighting.

## 2. Materials and Methods

### 2.1. Study Area

#### 2.1.1. Geographical Property

**Figure 1:**
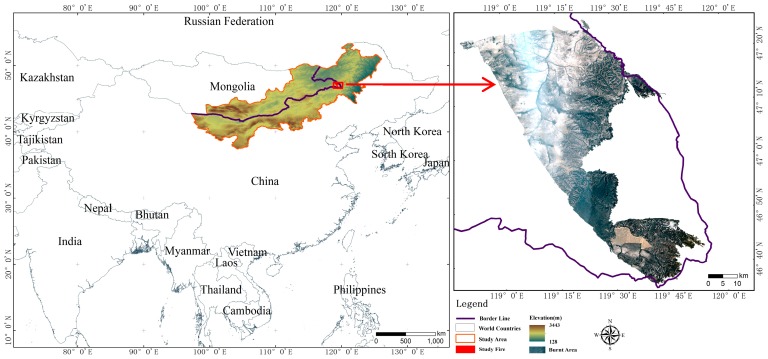
Map of the geographical location of the study area (**Left**) and the case fire location (**Right**).

The cross-border area of the Inner Mongolia Autonomous Region and Mongolia were selected as the study area (Figure 1). It is located in the south of the Mongolian Plateau, which lies between 37°21′–50°17′ N and 97°13′–122°44′ E, has a typical continental climate and from east to west is followed by the meadow steppes, typical steppes, and desert steppes area. The average lowest temperature is −18 to −22 °C, the average highest temperature is 19–22 °C and the annual average rainfall is 200–300 mm. It has the characteristic of obvious seasonal change and mostly gale-force wind days in winter, controlled by the Mongolian high pressure, cold and dry, with the rainfall and heat happening during the same period in summer [26]. In addition, the study area is located in the China and Mongolia border area, therefore it renders firefighting work more difficult. According to the results of previous studies on the temporal and spatial distribution of grassland fires in the Mongolian Plateau, the China-Mongolia border regions have widely distribution grassland fires in the Mongolian Plateau [27,28]. Among them, Khalkhgol, Matad, and Erdenetsagaan in Mongolia and East Ujimqin Banner in China are the area with the highest frequency of grassland fires.

This case of grassland fire exactly occurred in Khalkhgol in Mongolia adjacent to the East Ujimqin, Arshaan, New Barag left and New Barag right Banner in the Inner Mongolia Autonomous Region. The cases of grassland fires occurred in the meadow grassland area, a large of grassland regions, which is sparsely populated, with a higher and thicker grass and many units of fuels on the ground. In particular, natural climate conditions such as drought, gale wind days and strong sunshine in spring and autumn provide the necessary conditions in this area that cause a frequent occurrence of grassland fires [29]. All of these conditions have caused it to become one of the most serious areas for grassland fires in the Mongolia Plateau.

#### 2.1.2. News Report in Real-Time for the Case Fire

**Figure 2:**
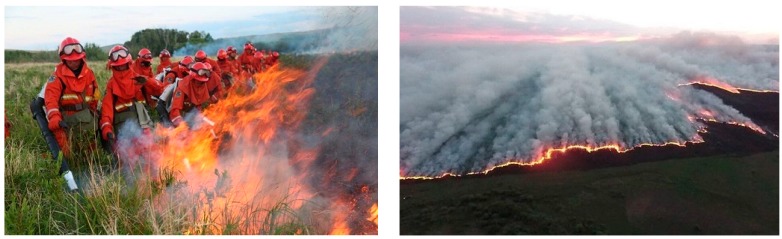
Scene of grassland fire in China-Mongolian border regions on 19–21 April, 2016.

Xinhua Net reporters Wu, Wang, and Liu reported on April 22st, 2016 that “The grassland fire in the China-Mongolia border area spread 4 days and extended the 200 km^2^”. The report said that the Armed Police Detachment of Forest in Hinggan League in Inner Mongolia used “fire attack to the fire” tactics, cutting off the grassland fire burning which continuously spread for 3 days in Mongolia near the Chinese border at 01:15 on 22st (Beijing time). The total burning fire control line was 22 km long, at 07:20 on 22st (Beijing time). For days on end, the border area of Inner Mongolia Autonomous Region adjacent to Mongolia frequently was struck by a grassland fire from outside. Inner Mongolia Ecological and Agricultural Meteorological Center monitoring show that the grassland fire area reached 200 km^2^ in Mongolia near the border of Inner Mongolia at 08:00 on 21st. Forest-fire prevention intelligence of the Hinggan league in Inner Mongolia discovered the fire behavior in Mongolia on the afternoon of the 19th found that it spreading to the direction of Chinese border. It is also reported that in view of continuous spreading grassland fire in Mongolia near the China-Mongolia border, armed police of the forest detachment in Hinggan league were deployed along the border of China and Mongolia [30]. Figure 2 shows soldiers putting out the fire and an aerial view of the fire line.

### 2.2. Data Sources

#### 2.2.1. Himawari-8 Satellite Data

**Figure 3:**
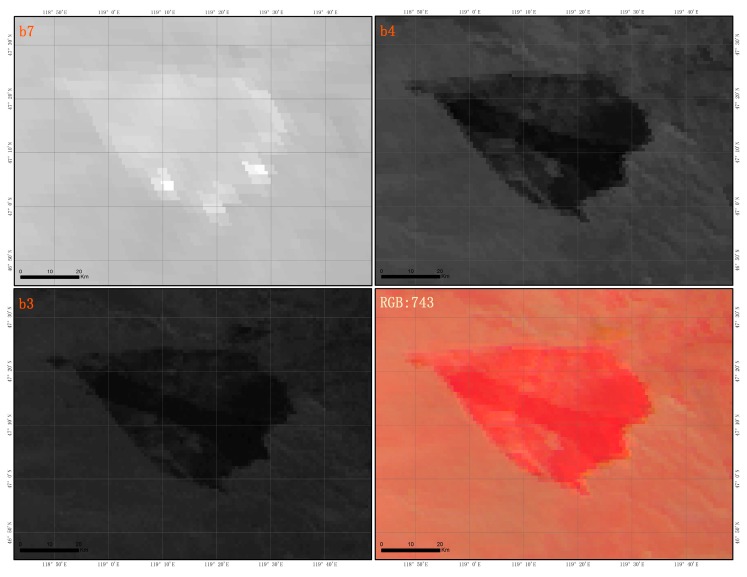
Grassland fire displays in bands 7, 4, 3, respectively, of AHI data and their RGB composite.

The Japan Meteorological Agency (JMA) launched Himawari-8 in October, 2014 and started its operation in July, 2015 [25]. It carries an AHI scanning five areas: Full Disk, the Japan Area, the Target Area and two Landmark Areas and in each 10-minute period, the AHI will scan the Full Disk once [31]. AHI sensor carried by Himawari-8 satellite has 16 channels, including a resolution of 500 m red channel, a visible green, blue and near-infrared channel with a resolution of 1000 m, and the remaining 12 channels have a spatial resolution of 2000 m, included in mid-infrared and thermal infrared. The MIR 3 µm channel is close to the spectral maximum for radiative emissions observed for objects radiating at temperatures found in fires, and in regions of low solar and terrestrial radiation [23]. Wooster demonstrated that MIR can be used to estimate the entire radiant energy from fire [32], so the band 7 of the AHI data is utilized to monitor thermal anomalies. The NIR channel has a relatively higher reflectance over vegetation in comparison to the Red, Green and Blue channels [23], therefore, spatial resolution of 1000 m band 4 with used to distinguish the change of vegetation spectrum between the burned zone and the no burned zone to extract the burned area, the burned zone pixel color in band 4 appears darker than the background [33]. In band 7, 4, 3 color synthesis image the burning fire pixel appeared the bright red color, the burn area and the smoke were respectively appeared the dark red and gray purple color (Figure 3). In this study, we will collect a total of 318 effective images, from 05:40 on 19th to 13:20 on April 21st 2016.

#### 2.2.2. Data of Evaluating for Monitoring Results

The MODIS Thermal Anomalies/Fire products are primarily derived from MODIS 4- and 11- micrometer radiance. The fire detection strategy is based on absolute detection of a fire, and on detection relative to its background. MOD14 is level-2 swath data provided daily at 1 km resolution. The Science Data Sets in this product include fire mask, algorithm quality, radiative power, and numerous layers describing fire pixel attributes. The Visible Infrared Imaging Radiometer Suite (VIIRS) sensor was launched aboard the Suomi National Polar-orbiting Partnership (NPP) satellite on October 28th, 2011 and on January 18th, 2012 cooler doors for the thermal sensor were opened. Within hours data were being retrieved and fire detection produced. The Global Fire (GFR) product uses FY-3 VIRR data to detect a hot target globally according to the features shown in both visible and infrared VIRR channels. The sub-pixel size and intensity of hot spot are evaluated by using infrared channel data. The hot spot intensity is based on the sub-pixel size and temperature of a hot spot, the resolution of the product is 1 km. We obtained the above three fire point product data to evaluate the burning fire point monitored by AHI.

The obtained wind field data was observed for each of the three hours from the Meteorological Station of China’s East Ujimqin, Arshaan, New Barag Right Banner and Mongolia’s Khalkhgol, Matad Banner around the case fire area, and the vegetation cover map was derived from the Vegetation Map of the Mongolian plateau provide by the Collage of Geography, Inner Mongolia Normal University. Given that the Landsat8/OLI data has a higher spatial resolution, the remote sensing data is more accurate, hence the pre- and post the case fire Landsat8/OLI images were obtained to extract the burn area, respectively on April 19th, and on May 4th, 2016. In addition, the case fire environmental elements information was extracted via the Landsat8/OLI image. Above data respectively to evaluate the result of speed and direction of the fire speared and result of burned area extracted by AHI data.

### 2.3. Methods

#### 2.3.1. Burning Fire Point Identification Algorithm

In order to improve the extraction accuracy of burning fire points, the Threshold Algorithm proposed by Chen [25] and Chathura [23] was used. Then, it was combined with the AHI data band 7, 4, and 3 after the RGB color synthesis by visual interpretation method to extract the burning fire. The specific steps are as indicated in the following Equations (1)–(4) [25].

First, to calculate the brightness temperature of the background pixel, initially, a 5 × 5 pixels kernel is used to calculate the average brightness temperature, $T_{b7\mathrm{bg}}=\mathrm{mean}(T_{b7})$ (the kernel of 5 × 5 pixels cloudless pixels are less than 20%, will incrementally expanded up to 7 × 7, 9 × 9, ..., 51 × 51. If the standard still cannot be reached, the pixels are excluded from the calculation and marked as a non-fire pixels). However, previously this calculation will need to have identified and removed clouds, water bodies and high temperature suspicious fire pixels. The conditions for the identification of high temperature suspicious pixels are as defined as follows in Equation (1):

(1) $$T_{b7}>T_{\mathrm{th}}\;\mathrm{or}\;T_{b7}>T_{b7\mathrm{bg}}+\Delta T_{b7\mathrm{bg}}$$

where $T_{b7}$ is the brightness temperature value of the band 7 and $T_{\mathrm{th}}$ is threshold value of the band 7. By default sum of the average of all the pixels in the kernel and the corresponding double of standard deviation can be used. $T_{b7\mathrm{bg}}$ is represents the band 7 average brightness temperature of the same land use in the kernel. $\Delta T_{b7\mathrm{bg}}$ is the difference between the suspicious fire pixel and the background brightness temperature and can be expressed as 2.5 times the standard deviation of pixels of the same land use type.

Secondly, for the confirmation of a fire pixel, if the pixel satisfies the following conditions, the pixel can be initially identified as a fire pixel, Equation (2):

(2) $$T_{b7}>T_{b7\mathrm{bg}}+a\times \delta T_{b7\mathrm{bg}}$$

where *δT*_b7bg_ is brightness temperature value of the band 7 in the kernel; a is a background coefficient, this coefficient varies with different regions and time; in this paper study area it can be set as 3.

Next, to confirm the reliability type of a fire point, Equations (3) and (4):

(3) $$f_{f}=T_{b7}>T_{Tb7\mathrm{bg}}+T:firepixels$$

(4) $$f_{pf}=T_{b7}<T_{b7\mathrm{bg}}+T:possible\;firepixels$$

where $f_{f}$ is fire pixels; $f_{pf}$ is the possible fire pixels; T is the threshold value of fire point reliability that can be used after the fire point has been identified and all pixels of the same type of land use 3 times the standard deviation in the kernel.

**Figure 4:**
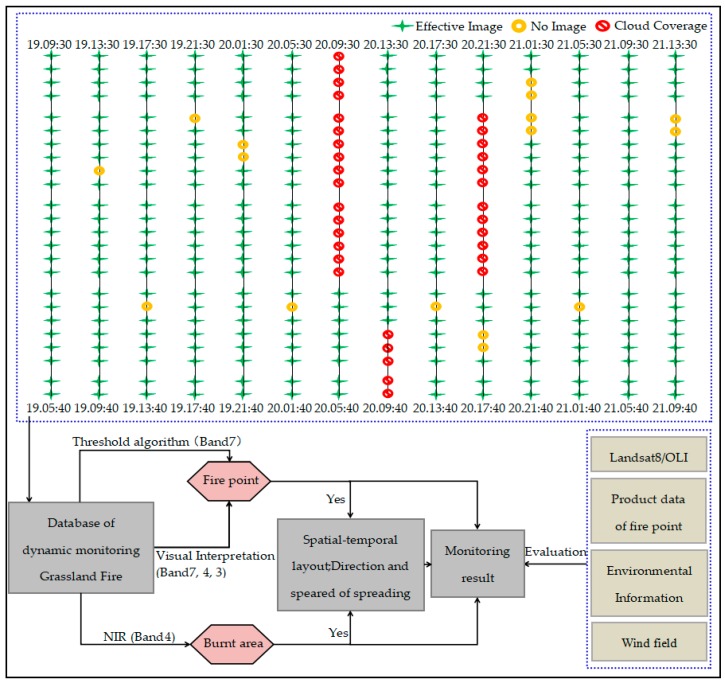
Methodology workflow. Remote sensing data sources are shown with dotted boxes.

Next, a Visual interpretation of the fire point is performed by band 7, 4, and 3 through RGB color synthesis, and the burning fire point is identified by extracting the bright red pixel (Figure 4). Finally, pixels that satisfy the above three requirements at the same time will be defined as the burning fire point, condition is as follows:

(5) $$\mathrm{Firepoint}=f_{f}/f_{\mathrm{pf}}\cap V_{i}$$

where *V_i_* represents the burning fire points monitored by visual interpretation.

#### 2.3.2. Spreading of Grassland Fire Analysis

When calculating the spread speed of grassland fire, the longest distance algorithm is used to calculate the longest distance between the period of fire point at the beginning and the fire point at the end. The calculated equation is as follows:

(6) $$S_{\nu }=\frac{d_{\mathrm{ij}}}{t}(d_{\mathrm{ij}}>d)$$

where *S_v_* is the spread speed of the grassland fire. Within a period of time, the term *d_ij_* is the distance between point *i* and point *j*, point *i* is the start point of the fire, the point *j* is the end point of the fire and the *d* is distance the from *i* point to any point of end point; and *t* is duration of the grassland fire spread time in this time period.

#### 2.3.3. Grassland Fire Burned Area Extraction

(1) Extraction of burned area from AHI data

The extraction of the burned area pixels by satellite remote sensing data is conducted according to the change of surface radiation characteristics before and after the fire. After burning, the spectral characteristics of grassland vegetation significantly changed after being burned due to the damage of the chlorophyll cells and the spectral changes are mainly reflected in the rapid decline of the ground reflectance in the near infrared channel. The near infrared channel of AHI data is sensitive to changes in vegetation, and the burned areas show dark black in the band 4 compared with the background pixels (Figure 4). Therefore, we will use the visual interpretation based on band 4 to monitor the dynamic change of the burned area in this paper.

(2) Extraction of burned area from Landsat8/OLI

The burned area was extracted by combining a Differenced Normalized Burn Ratio (dNBR) and visual interpretation methods. The dNBR index was used to calculate the difference NBR values from two same-scene image data of before and after the burn. The calculated Equation is as follows Equations (7) and (8) [34,35]:

(7) $$\mathrm{dNBR}={\mathrm{NBR}}_{pre}-{\mathrm{NBR}}_{post}$$

where ${\mathrm{NBR}}_{pre}$ and ${\mathrm{NBR}}_{post}$ are the NBR value of before and after the burn.

The NBR value is calculated as follows:

(8) NBR=(NIR−MIR)/(NIR+MIR)

where NIR is a band 5 reflection and MIR is a band 7 reflection of Landsat8/OLI data. In addition, the normalized Burn Ratio (NBR) index’s theoretical range of values is [1, −1].

#### 2.3.4. Evaluation for Monitoring Results

Wind is the dominant factor that affects the speed and direction of grassland fire burning, however, it mainly affects three main aspects. Firstly, it accelerates the evaporation of combustible water, promotes drying, especially a hot and dry wind, and it helps burning as a direct result of a decrease in combustible moisture. Secondly, it replenishes the oxygen in the fire to make the fire stronger and burn faster. Thirdly, it allows a small fire to expand, and can allow it to resurge, especially after a fire has broken out, as fires spread mainly in the downwind direction. At this time, if a gale were encountered, it would cause a large area of grassland fire to burn. In addition, the grassland type of the fire field is an important factor that affects the burned area, and the quantity and continuity of the grass or not will determine the burning of the grassland fire. The environmental information around the burning area, such as roads, residential spots, rivers and fire isolation belts, will affects the path of the development of the grassland fire.

Therefore, we verified whether the fire point monitored by the product data and monitored by the AHI data conformed to the fire point position and time in between. Each three hours the wind field data and environmental information was compared with the spreading speed and direction of the grassland fire monitored by the AHI data and it was observed whether the two matched. In addition, the overlying burned area was extracted from AHI data band 4 and Landsat8/OLI data to calculate the precision.

#### 2.3.5. Methodology Workflow

The entire study consists of four steps: (1) monitor the burning fire point; (2) estimate the speed and direction of the spreading grassland fire; (3) extract the burned area; (4) evaluate the monitoring result (Figure 4). The details are as follows: first, obtained the AHI image data, and preprocessing of the AHI image is performed, include geometric correction (choosing the Geographic WGS84 Projection) and cropping, which will then be used to generate a dynamic monitoring database of grassland fires in the study area. Then combining with the Threshold Algorithm and visual interpretation method, the burning fire point is extracted from the band 7, 4, and 3. Using the changes of the burned area display on the band 4 to visually interpret the burned area. By the optimized in time series of the burning fire point and burned area, the dynamic changes of the fire points are used to calculate the speed and direction of the fire spread through the maximum distance method. Secondly, we obtain the fire point product data, to evaluate the burning fire point monitored by AHI, then obtain the wind direction and speed data to evaluate the calculated speed and direction of the fire spread result. Finally, Landsat8/OLI data was used to extract the environmental information data of the area in which the fire took place and entire burned area, to evaluate the result of the burned area extracted by AHI data.

## 3. Results

### 3.1. Analysis of the Result of Dynamic Monitoring the Burning Fire Point 

**Figure 5:**
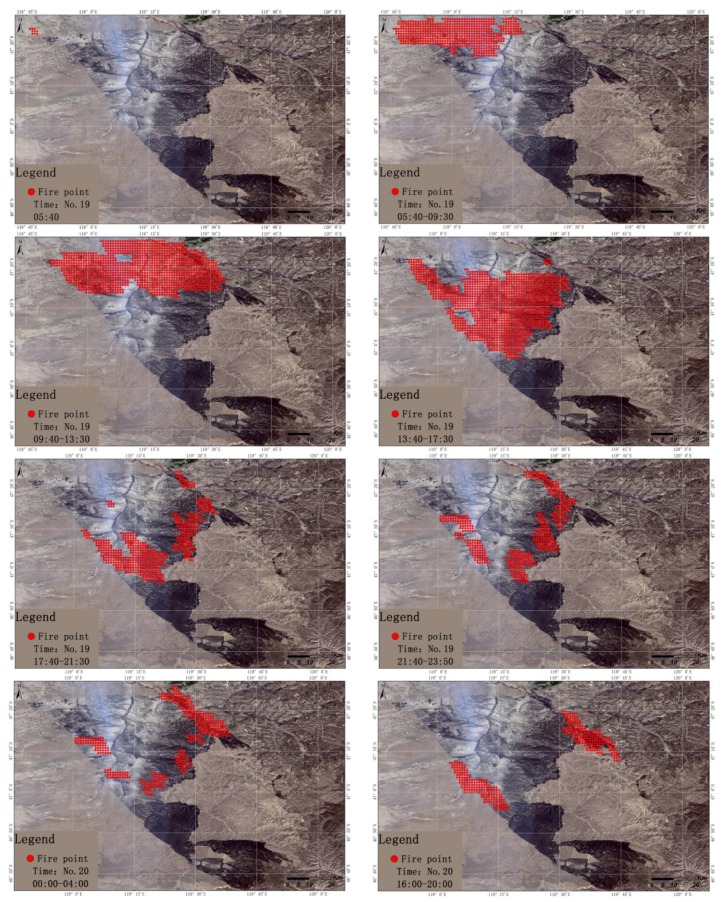

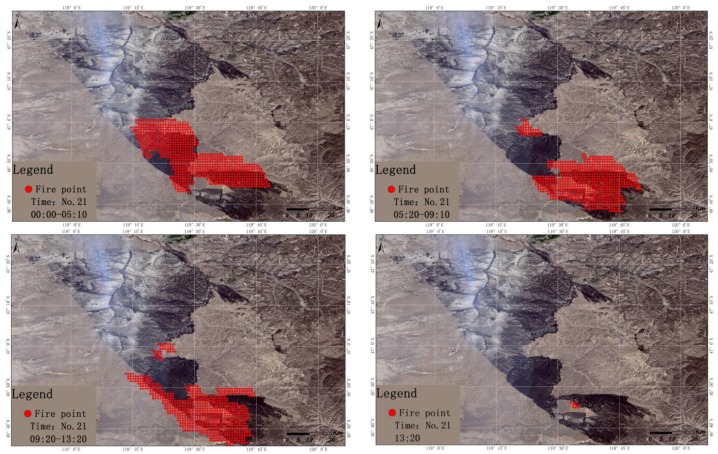
Dynamic monitoring of burning fire points in a continuous time period from 05:40 on the 19th, to 13:20 on April 21st, 2016. The background is the Landsat/OLI image of May 4, 2016 that shows the burned area.

Figure 5 displays the entire process of the case fire dynamics. It can be seen from the figure that the first fire point detected in the grassland fire was located at 47°23′31′′ N and 118°46′17′′ E within the territory of the Khalkhgol in Mongolia, at 05:40 on April 19th, 2016. From the extracted fire points, we can see that from 05:40 on the 19th to 00:50 on the 20th the number of burning fire points displayed a rapidly increasing trend, in which the number of fire point pixels increased to 314 at 13:50 on the 19th. However, from 01:00 to 23:40 on the 20th the number of burning fire points gradually decreased, showing a state of extinction, but scattered residual fires were still burning. Starting at 23:50 on the 20th, the number of fire points increased significantly, once again forming a large area extending from northwest to southeast of the grassland fire and lasting until 11:50 on the 21st. Among them, from 16:10 to 19:40 on the 20th, the burning fire points continued to appear on the border of Arshaan, and burning fire pixels were detected in East Ujimqin at 12:10 to 13:10 on the 21st in China.

The monitoring results show that the grassland fire case that occurred in Mongolia crossed the border twice into China, leading to the formation of a cross-border grassland fire in China. Since then, only nine fire pixels were detected at 13:20 on the 21st, roughly at the location of 46°45′42″ N, 119°34′5″ E. In the ensuing images, no fire information was detected, therefore the grassland fire extinction time was about 13:30 on April 21st, 2016. In the entire grassland fire process, a total of 770 burning fire pixels were detected without counting the repeating time point, with a total range of 46°35′3′′–47°25′2′′ N, 18°46′13′′–119°44′2′′ E.

### 3.2. Analysis of the Speed and Direction of Grassland Fire Spread 

**Figure 6:**
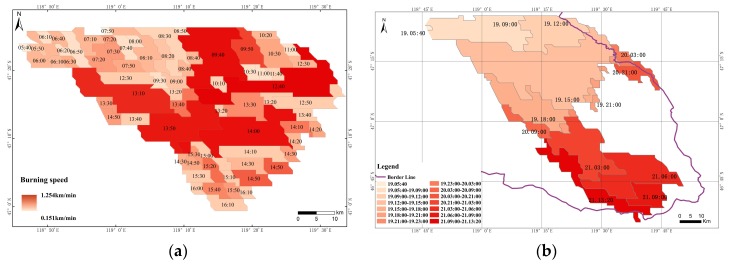
(**a**) is the ten minute spread speed of grassland fire during from 05:40–16:10 on April 19, 2016; (**b**) display the dynamic changes of a grassland fire burned area over a continuous time period.

The spread of grassland fire can be divided into three directions. Specifically, from west to east from 05:40 to 12:00 on the 19th, and from 12:00 to 21:00 on the 20th, generally from north to south and partial extending eastward. While the entire path of the 21st burned from northwest to southeast, the direction of burning changes with the change of wind speed in real-time. In the estimation rate of spread in this grassland fire (Figure 6a) the spread of the fire points after 16:10 on the 19th spread every ten minutes without an obvious outward extension. Hence, only the rate of spread of the natural combustion part was estimated. The highest burning speed of the grassland fire was 20.9 m/s, the lowest speed was 2.52 m/s and the average speed was about 12.07 m/s, in this case of the grassland fire. The spread of the grassland fire from the starting period to 09:00 (7.7 m/s) was slower than the later stage. The spread speed was higher at 09:40, 09:50, 12:40, 13:10, 13:50 and 14:00, with an average speed of 19 m/s and a larger area of expansion. While during 14:00 to 16:10 the rate of fire spread at an average speed of 9.5 m/s was relatively reduced when compared to the above period.

### 3.3. Analysis on the Result of Dynamic Extraction of a Burned Area

In the past, using the Cellular Automaton (CA) simulation to study the combustion process of grassland fires, assuming that no one extinguished the fire, then the burned area of the grassland fire would show a fan-shaped diffusion to the southeast [36]. The burned area of the grassland fire from 05:40 on the 19th to 09:00 on the 20th of April 2016 displayed a fan-shaped, at 46°56′57″–47°26′19″ N, 118°45′46″–119°33′32″ E. However, most of the burnt area at this time was within the border of Mongolia, indicating that this grassland fire may not be effectively controlled within the territory of Mongolia and could not be extinguished until it crossed the border in China (Figure 6b). The burned area reached 1620.80 km^2^ on the 19th, of which the largest burned area was between 12:00 to 15:00 on the 19th, with an area of 650.65 km^2^. Since the fire on the 20th consisted of the burning of scattered fires after the large-scale fire on the 19th, the burned area on the 20th was smaller with a total burnt area of 167.11 km^2^. In addition, And the fire on the 21st was developed from the remaining fire on the 20th to a large fire, extending from northwest to southeast, with a burnt area reaching 920.38 km^2^. The entire range of fire marks ranged from 46°35′–47°26′ N, 118°48′–119°54′ E with a burnt area of 2708.29 km^2^. Through the above result, we find that the dynamic changes of the burned area and dynamic changes of the burning fire point correspond to each other in time and position, and were basically the same.

### 3.4. Analysis on the Results of the Grassland Fire Monitoring Evaluation

**Figure 7:**
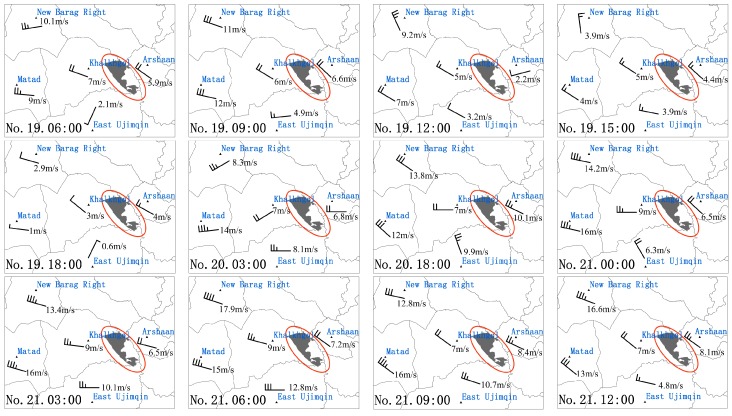
The wind field maps of the five meteorological sites, once every three hours (East Ujimqin, Arshaan, New Barag Right, in China and Khalkhgol, Matad, in Mongolia.).

By observing the wind field maps of the five meteorological sites in East Ujimqin, Arshaan, New Barag Right in China and Matad, Khalkhgol in Mongolia on April 19, 20 and 21, it can be found that except for the wind direction at 03:00 on the 20th, it was usually a westerly or northwesterly wind, with more northwesterly winds, this is consistent with the change of the spread route of the grassland fire from west to east and from northwest to southeast (Figure 7). Comparing the wind speed, it can be found that the New Barag Right and Matad had a particularly high wind speed of up to 12 m/s at 09:00, 12:00 on the 19th, which caused an increase of the burned area from 12:00 to 15:00 on the 19th. Although the wind speed on the 20th was larger than on the 19th, because the fire on the 20th was in a semi-extinguished or extinguished state, no burning fire point increased. However, the wind speed on the 21st reached a maximum of 17.9 m/s and the wind speed was high all day. As a result, the fire that caused the semi-extinguished fire started to rekindle, leading to the re-formation of a large fire again. This is consistent with the rapid increase in the number of fire points on the 21st monitored by AHI data and large fire areas.

**Figure 8:**
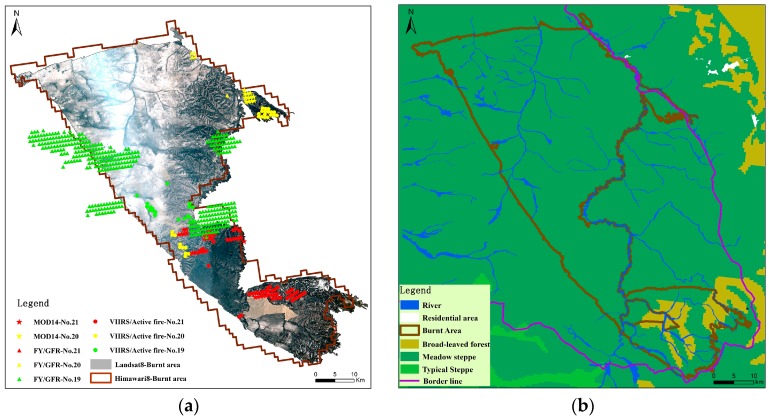
(**a**) is a fire product data distribution and burnt area extraction from the Landsat8/OLI image; (**b**) display the environmental information of case fire regions.

Based on the three days of product data of fire point were obtained during the 19–21 of April 2016. The data included the Global Fire Point product data MOD14 of Terra sensors equipped with MODIS satellites on April 20, 21 and VIIRS sensor equipped with Suomi-NPP satellite and GFR data equipped with FY-3 Weather Satellite, on April 19–21 (data introduced in Section 2.2.2), the fire points on the 19th were mainly concentrated in the northwest part of the burned area, while on the 20th in the northeast part, and on the 21st in the central and southern part (Figure 8a). This result is not only consistent with the burning time of the fire, but also with the timing of the fire points detected by the AHI data.

Judging from the type of the grassland in the burned area, this grassland fire occurred in the meadow steppe area. The vegetation types mainly includeded *Stipa baicalensis* Roshev, *Aneurotepidimu chinense*, *Arundinella anomala* Steud, *Calamagrostis epigeios*, and Chaihu in the meadow steppe area. Due to the hydrothermal conditions, vegetation grows higher and thicker. The characteristics of the fuel material continuity and low ignition point in the meadow steppe just meets the requirements of the combustibles in this fire. This fire located in the China-Mongolia Border regions, also occurred during the windy season in April, which increased the difficulty of firefighting work (Figure 8b). Comparing the distribution of the burned area and the surrounding environment, we found that the burned areas are mainly distributed along the river and the border line, which is in good agreement with the actual situation. Due to the different spatial resolution of images, the burned area extracted by Landsat8/OLI was 2448.13 km^2^, while according to the AHI data it was 2708.29 km^2^.

## 4. Discussion

Grassland fire surveillance is an important aspect of fire management, as such near real-time satellite monitoring systems will provide great benefits to fire agencies for grassland fire management and response [23]. However, most studies are focused on the spatial and temporal distribution of grassland fires over a long period of time and over a large scale, therefore, the studies of monitoring the burning fire point, estimating the direction and speed of spread and the dynamic extraction of a burnt area can provide technology for obtaining timely information on fires for the fire prevention work of fire prevention departments. In this paper, the results of the combined band 7 Threshold algorithm with visual interpretation by band 7, 4, and 3 color synthesis to monitor the burning fire point agree with the results of Chen and use a 3.9 µm and 11 µm channel of AHI data through the Threshold Algorithm to extract the temperature information of the fire in 2017 [25]. In addition, the accuracy of fire point monitoring is higher than in Hally in 2016 using the band 7 brightness Threshold algorithm [24]. However, the method of visual interpretation by band 7, 4, and 3 color synthesis to extract the fire point compared with the Fatkhuroyan Method [22] that used Himawari-8 satellite by band 3, 4, and 6 color synthesis to monitor fire information, provides more obvious burning fire point, while the latter’s smoke characteristics are more obvious. Nevertheless, the more critical aspect in monitoring grassland fires is not only to extract the burning fire point, but also to monitor the dynamics of fire points and burnt areas, as well as the monitoring of the spread speed and direction. Hence, we monitored the entire process of the fire, including not only dynamically monitoring of the fire point, but also dynamically extracting the burned area and estimates of the direction and speed of the fire spread.

Except for Chathura [23], the results of using Himawari-8 in the field of grassland monitoring were compared with the result of monitored by MODIS to detect the fire point to test the accuracy of result, the over 80% accurate detection was achieved and the other researchers did not perform further verification of the monitoring results. Therefore, we used the wind field data taken at one time each three hours, the environmental information data of the area in which the fire took place, the fire product data, the burnt area extracted from the higher resolution Landsant8/OLI image data and the real-time news reports are used evaluate the results of entire the case fire monitoring. The results of monitoring grassland fire using AHI data are consistent with news reports and the real-time direction and speed of wind [30], because of the difference in spatial resolution, the total burned area exceeded 260.16 km^2^ compared with burned area extracted from Landsat8/OLI images. The amount of effective images of the AHI data in this case fire period is also large, so this monitoring result is also quite satisfactory. The limitation of this paper is estimation the speed of fire spread in Figure 6a, where the spread of fire points after 16:10 on the 19th has no regular extension outward every ten minutes, so the speed of burning of the entire case fire has not been calculated, and we only estimated the rate of spread of the natural burning part. AHI data have a high temporal resolution but a low spatial resolution, so it should be combined with high spatial resolution data in the future research, to further establish a rapid fire information monitor model, so as to extract fire information for up to the minute resolution.

## 5. Conclusions

In this grassland fire case, the burning time was between 05:40 on 19th to 13:20 on 21st April 2016, a total of burning two days and eight hours, and the total burnt area was 2708.29 km^2^. Owing to the change of wind direction and speed, the fire spread direction changed from west to east int the initial stage to extend from northwest to southeast. During this time, the border was crossed two times into Arshaan and East Ujimqin in China. The results of monitoring the burn spread from 05:40 to 16:19 on April 19th show that the maximum burning speed was 20.9 m/s, the minimum speed of 2.52 m/s, and the average speed was about 12.07 m/s. The evaluation monitoring results indicated that, the time and location of AHI data monitoring the burning fire point coincided with fire point product data, and more than the product data to monitor the number of fire points accurately. The speed of grassland fire spread agrees well with the real-time wind speed at the weather station. In addition, the changes in the route of spreading coincided with the current wind direction, as the surrounding environment and burnt area of Landsat8/OLI images shows.

Dynamic monitoring grassland fire based on Himawari-8 satellite AHI data using band 7, 4, and 3 can increase the temporal resolution by seconds and increase the spatial resolution to 500 m. AHI data can provide full play to satellite remote sensing high efficiency, along with the advantages of frequency observation. By analyzing fire images and fire monitoring information, the fire point location and the burnt area can be quickly and accurately obtained, the speed and direction of fire spread can be estimated, and the dynamic changes and duration of a grassland fire can be obtained. This research has a great significance with regards to monitoring the development of fire behavior and quick assessment, and provides an important basis for the fire prevention departments to take timely fire prevention measures.
